# First approach to distinguish between cardiac and arteriosclerotic emboli of individual stroke patients applying the histological THROMBEX-classification rule

**DOI:** 10.1038/s41598-021-87584-2

**Published:** 2021-04-19

**Authors:** Florian C. Roessler, Nicolas Kalms, Florian Jann, Andrè Kemmling, Julika Ribbat-Idel, Florian Stellmacher, Inke R. König, Marcus Ohlrich, Georg Royl

**Affiliations:** 1 Department of Neurology, Justus-Liebig-University Gießen, Klinikstraße 33, 35385 Gießen, Germany; 2 Department of Neuroradiology, Westpfalz-Klinikum, Hellmut-Hartert-Straße 1, 67655 Kaiserslautern, Germany; 3 Institute of Pathology, University of Lübeck and University Hospital Schleswig-Holstein, Campus Lübeck, 23538 Lübeck, Germany; 4 Research Center Borstel - Leibniz Lung Center, 23845 Borstel, Germany; 5 Institute of Medical Biometry and Statistics, University of Lübeck, Ratzeburger Allee 160 (House 24), 23562 Lübeck, Germany; 6 Department of Neurology, Sana Kliniken Lübeck GmbH, Kronsforder Allee 71-73, 23560 Lübeck, Germany; 7 Department of Neurology and Center of Brain, Behaviour and Metabolism, University of Lübeck, Ratzeburger Allee 160, 23538 Lübeck, Germany

**Keywords:** Stroke, Microscopy

## Abstract

Endovascular treatment of strokes caused by large vessel occlusion enables the histopathological investigation of the retrieved embolus, possibly providing a novel opportunity to contribute to the diagnostic workup of etiology and to define secondary prevention measures in strokes with uncertain genesis. We aimed to develop a classification rule based on pathophysiological considerations and adjustment to reference thrombi for distinction between cardiac and arteriosclerotic emboli and to validate this classification rule on a patient cohort. From 125 patients with stroke due to large vessel occlusion and thrombectomy, 82 patients with known etiology (55 cardioembolic and 27 arterioembolic strokes) were included. The corresponding emboli were histologically evaluated by two raters blinded to the etiology of stroke by means of a novel classification rule. Presumed etiology and classification results were compared. Agreement concerning cardiac emboli was 72.2% (95% CI: 58.4–83.5) for rater I and 78.2% (95% CI: 65.0–88.2) for rater II. Agreement concerning arteriosclerotic emboli was 70.4% (95% CI: 49.8–86.3) for rater I and 74.1% (95% CI: 53.7–88.9) for rater II. Overall agreement reached 71.6% (95% CI: 60.5–81.1) for rater I and 76.8% (95% CI: 66.2–85.4) for rater II. Within the limits of generally restricted accuracy of histological evaluations, the classification rule differentiates between cardiac and arteriosclerotic emboli of acute ischemic stroke patients. Further improvement is needed to provide valuable complementary data for stroke etiology workup.

## Introduction

Ischemic stroke is a major burden for healthcare. Increasing efforts have been made to improve secondary prevention, as 6.2% of minor strokes will suffer from a second possibly devastating stroke within one year, despite a continually applied treatment^1^. The recent advances in intraarterial neuroradiological intervention techniques as acute stroke treatment have enlarged the number of patients who have a good outcome from an otherwise devastating ischemic stroke caused by a larger artery occlusion^2^. The occlusion is commonly due to an embolus. The common site from which this occluding embolus originates is a stenosis of an internal carotid artery, the left atrial appendage, or the left ventricle. However, in a relevant portion of patients a so-called ESUS (“embolic stroke of unknown source”) is the result of a diagnostic workup, usually including cerebral CT or MRI, cerebrovascular imaging by CT or MRI angiography or ultrasonography, echocardiography and ECG monitoring^3,4^. These patients are dismissed with uncertainty regarding the optimal secondary prevention, as the undifferentiated approach of treating ESUS patients with novel oral anticoagulants has not been shown to be beneficial in randomized studies^5,6^. Thus, the diagnostic workup of stroke etiology remains an indispensable cornerstone in order to decrease the mortality and morbidity of patients with ESUS or competing stroke causes.

Recently, diagnostically relevant information has been introduced into this diagnostic field by examining the histology of emboli^7–16^. This approach uses thrombus material obtained from thrombectomies and thus takes advantage of the fact that in these patients, an embolic cause of stroke is very probable, and that the causing thrombus material is accessible. However, since experience in this field is limited, the optimal methodological strategy of examining this material is not known^10^.

The THROMBEX trial is a prospective, blinded trial performed in Lübeck and Gießen, Germany. The histological features of emboli extracted from acute ischemic stroke patients are examined and correlated with anamnestic, clinical, and imaging data as well as with periprocedural parameters.

As a first step, we attempted to provide a novel opportunity to contribute to the diagnostic workup of stroke etiology. This requires a differentiation between cardiac and arteriosclerotic emboli, which are the most frequent causes of proximal vessel occlusion of acute ischemic stroke patients. For this purpose, the following pathophysiological considerations were taken into consideration:

In the course of many cardiac diseases, the endocardium is frequently damaged. There is evidence that endothelial dysfunction is the primary underlying pathology that directly triggers clot formation with the side effect of atrial fibrillation and hemodynamic changes in the auricles^17,18^. Generally, separation thrombi originate in the area of endothelial damage^19–24^. Longer dwell times in the heart cavity caused by associated atrial fibrillation and reduced blood flow velocity enable increased clot organization with progressive cell death, further local thrombin activation, accumulation of fibrin and increased cross-linking. The resulting clots (described as “white clots” due to their low concentration of hemoglobin) contain dense fibrin nets, many and widely distributed platelets, as well as many shattered neutrophils and red cells^19,20,22,24,25^. Those separation thrombi become harder and more resistant before they are carried away by the blood flow causing a stroke.

Agglutinative thrombi do not exclusively result from hemodynamic impairment around reduced blood flow velocities. They also emerge in areas of high blood flow velocities as the tail part of a mixed-thrombus or from clotting processes in the slipstream of a stenosis. Usually, the head section of the mixed-thrombus is a separation thrombus firmly attached to a damaged arterial vessel wall^20,26–28^. They are torn away soon after formation by fast streaming blood and flow turbulences. The histological composition of agglutinative thrombi is smooth and loose with only little organized structure. In consequence, these “red clots” have a fine fibrin net, few and locally restricted platelets, and many intact neutrophil granulocytes and red cells. Due to these reasonable and profound results of prior investigations, we hypothesize that:

1. Cardiac emboli comply with separation thrombi with dense fibrin nets, many platelets, and small proportions of intact neutrophil granulocytes and red cells. These clots are dense, compact, rigid, stable and appear older.
2. Arteriosclerotic emboli usually result from agglutinative thrombi with only fine fibrin nets, few and locally restricted platelets, masses of red cells and high proportions of intact neutrophils and red cells. These clots are smooth, unstable, and their structure is poorly organized.

The goal of the present work is the development of a histological classification rule that is based on these hypotheses and its validation on a cohort of stroke patients.

## Methods

The THROMBEX trail was approved by the local ethics committees in Lübeck (University of Lübeck, reference number: 11-257) and Gießen (Justus-Liebig-University Gießen, reference number: 281/13). All patients gave written informed consent prior to inclusion in the study. All experiments were performed in accordance with the Declaration of Helsinki.

### Acquisition of thrombotic emboli and determination of stroke etiology

One thrombotic embolus was collected from each of 125 different acute ischemic stroke patients who underwent thrombectomy. In all cases, mechanical thrombectomy was performed by a standardized coaxial procedure with stent retriever systems (Trevo XP, 4 × 20 mm or 6 × 25 mm, Stryker, Fremont, USA).

105 emboli were removed from the anterior and 20 from the posterior circulation.

**Figure 1:**
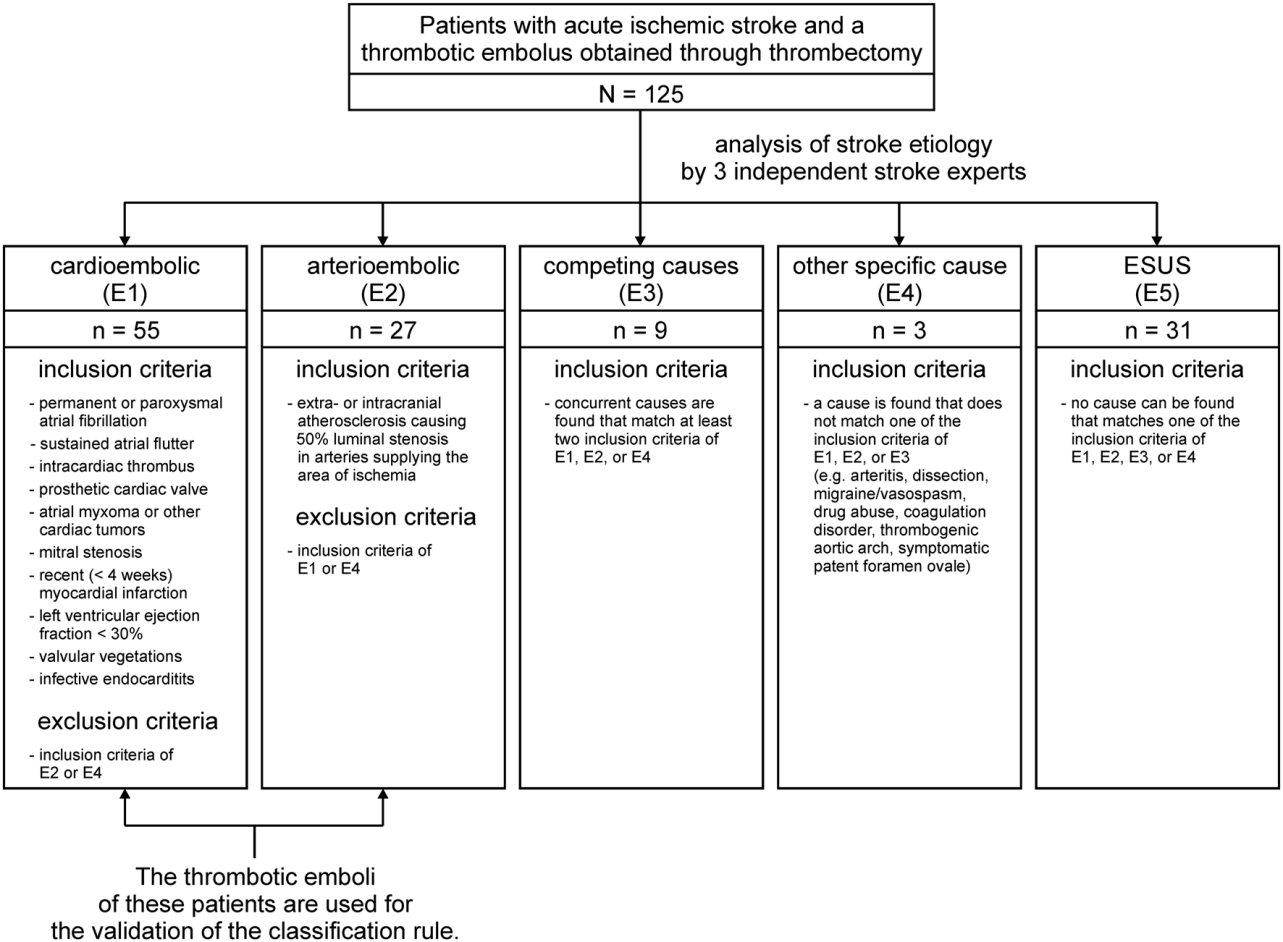
Selection process of suitable thrombotic emboli for the validation of the classification rule. Stroke etiology was evaluated for 125 patients with acute ischemic stroke who underwent thrombectomy. The evaluation was independently performed by three stroke experts and followed the ESUS criteria of the Cryptogenic Stroke/ESUS International Working Group. ESUS: embolic stroke of undetermined source.

Following the ESUS criteria proposed by the Cryptogenic Stroke/ESUS International Working Group^3,29^ the underlying cause of stroke was evaluated independently by three stroke experts (board certified neurologists with at least one year experience in treating stroke patients) (Fig. 1). For this purpose, the detailed medical history and all diagnostic results had to be fully available. Instrumental investigations should at least include CT or MRI of the brain, 12-lead ECG, a 24-h Holter ECG, precordial or transesophageal echocardiography, and imaging of both the extracranial and intracranial arteries supplying the brain by catheter, MR, or CT angiography, or cervical and transcranial duplex sonography. Furthermore, patients who were younger than 55 years received a coagulation analysis and vasculitis serology. Only emboli from patients with cardioembolic or arterioembolic strokes unequivocally specified in the same way by all three stroke experts were included for the validation of the classification rule. Thus, 55 emboli of patients with a cardioembolic and 27 emboli of patients with an arterioembolic ischemic stroke were analyzed. Clinical characteristics of the two groups are listed in Table 1.

**Table 1:** Baseline characteristics of patients included in the validation analysis.

Baseline characteristics	Cardioembolic (E1) N = 55	Arterioembolic (E2) N = 27
Years of age [median (min; max)]	72 (44; 88)	67,5 (45; 81)
Female sex [%]	50,9	48,1
**Vascular risk factors**		
Arterial hypertension [%]	76,4	81,5
Diabetes mellitus [%]	25,5	29,6
Coronary artery disease [%]	25,5	22,2
Atrial fibrillation [%]	87,3	3,7
Hyperlipoproteinemia [%]	20,0	22,2
History of smoking [%]	16,4	51,9
**Physical state on admission [median (min; max)]**		
Body mass index [kg/m^2^]	25,7 (19,1; 53,6) n = 52	27,7 (20,5; 36,1) n = 26
Systolic blood pressure [mmHg]	144 (97; 240)	165 (119; 190)
Blood glucose [mg/dl]	114 (68; 180)	113,5 (97; 196)
NIHSS	14 (4; 37) n = 53	14,5 (4; 37)
**Laboratory data on admission [median (min; max)]**		
Platelet count [/nl]	218 (98; 373)	245 (142; 430)
Hematocrit [%]	39 (21; 46)	42 (33; 49)
**Premedication**		
Antiplatelet therapy [%]	49,1	29,6
Anticoagulation [%]	20,0	3,7
**Occlusion site**		
On the right: ICA, MCA, carotid T occlusion [%]	47,3	48,1
On the left: ICA, MCA, carotid T occlusion [%]	38,2	44,4
Basilar artery [%]	14,5	7,4
**Thrombolytic therapy**		
None [%]	21,8	22,2
Bridging with Abciximab [%]	29,1	25,9
Systemic application of rt-PA [%]	49,1	51,9
Local application of rt-PA [%]	10,9	7,4
**Course of thrombectomy [median (min; max)]**		
Duration: Symptom onset–recanalization [min]	204 (87; 625) n = 47	229,5 (135; 575) n = 16
Recanalization time [min]	52 (9; 169) n = 53	48,5 (17; 153)

For continuous variables, the median and the minimum and maximum values are indicated. Nominal variables are presented as percentage. In cases of missing data, the number of available data is specified. NIHSS: National Institutes of Health Stroke Scale. ICA: internal carotid artery; MCA: middle cerebral artery.

### Acquisition of reference thrombi

11 clots of certain origin served as reference thrombi. Five clots were withdrawn out of left-sided heart cavities during cardiosurgical interventions. They were considered to be cardioembolic clots. Six clots represented arterioembolic clots as they were extracted from coronary arteries during cardiac catheter examinations.

### Histological examination

All samples were formalin-fixed (4% buffered formalin, BÜFA, Hude, Germany) and paraffin embedded (FFPE, Paraffin, Leica Microsystems, Wetzlar, Germany).

2 µm thick slices were cut from the paraffin blocks by microtome and placed on Super Frost glass slides (Menzel, Braunschweig, Germany). Hematoxylin and eosin (HE) stains (Merck, Darmstadt, Germany) and Elastica-van Gieson (EvG) stains (Merck, Darmstadt and Waldeck/Chroma, Münster, both Germany) were carried out. Immunohistochemistry was performed using the PostBlock HRP-Polymer and ZytoChem Plus Kit (both Zytomed, Berlin, Germany). The CD61 (Zytomed, Berlin, Germany) antibody was diluted 1:250 and pretreated by steamer. Serial sections were stained and arranged on a single microscope slide. Thus, all clots were screened over a wide range before a representative section was selected for further evaluation. The histological cross sections were captured with 200-fold original magnification. Only fibrin net was pictured with 400-fold original magnification. Two raters using Olympus BX50 microscope or Carl Zeiss Axioskop 40 microscope both with fluorite objectives with plano-correction independently examined all hematoxylin and eosin (HE), Elastica-van Gieson (EvG) and CD61 stains. Both raters were blinded concerning the pathogenesis of the clots, their total number, the clinical diagnosis of the stroke patients and their demographics and treatment. For technical reasons, rater I could only evaluate 81 of the 82 emboli that were extracted from the stroke patients.

**Figure 2:**
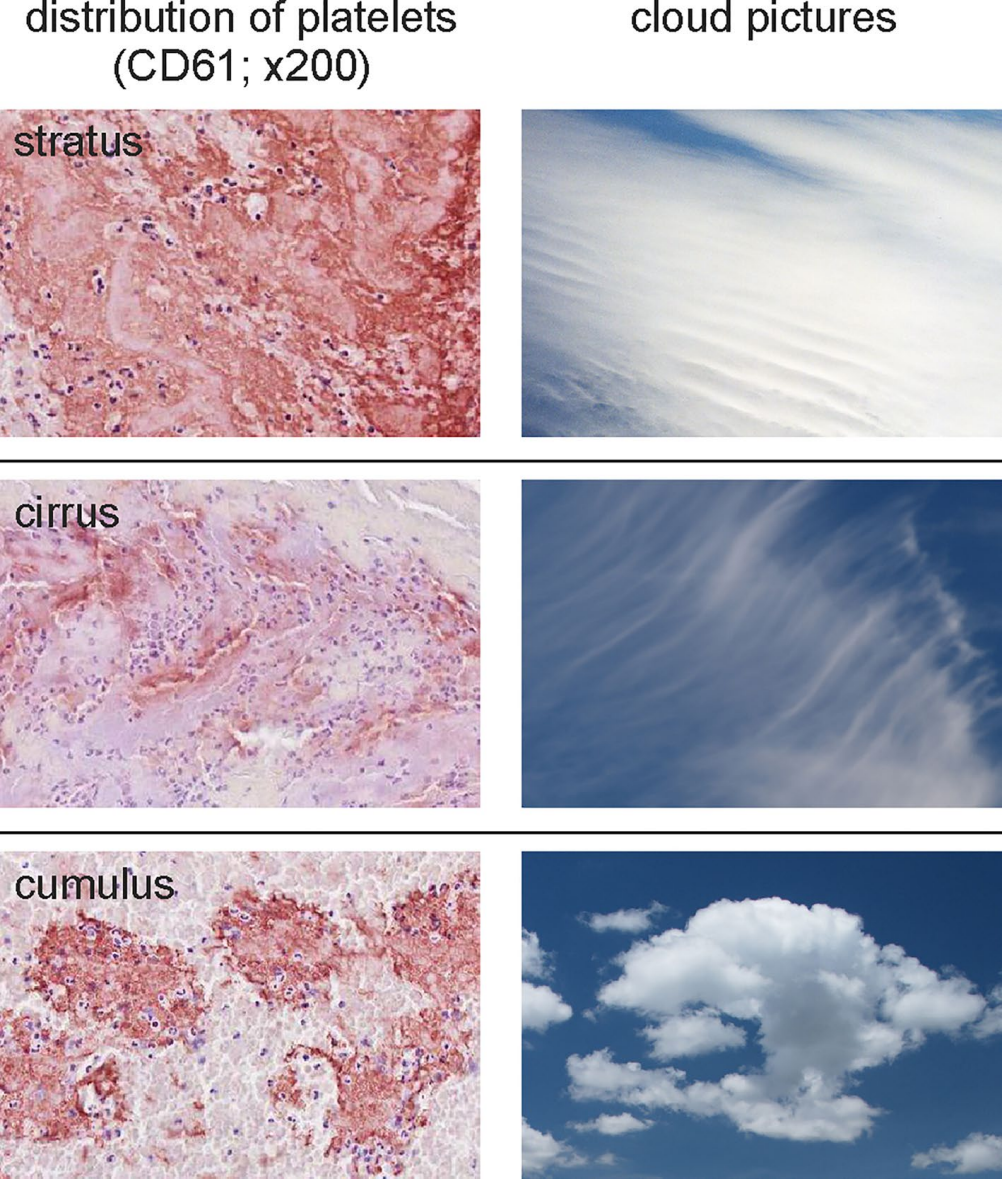
Description of different platelet distributions used by the THROMBEX clot classification. In analogy to basic meteorological cloud shapes (right side) used by the World Meteorological Organization published in the International Cloud Atlas^30^ we described platelet distribution within clots (left side) as “stratus”, “cirrus”, and “cumulus”. Following the wording of the International Cloud Atlas, in a stratus distribution platelets occur as a merged layer with a uniform appearance or in the form of ragged patches. A cirrus distribution is characterized by detached clusters in the form of delicate filaments or narrow bands, having a hair-like appearance. Cumulus-like arranged platelets appear as detached and dense clusters with sharp outlines. They may be in the form of domes or towers. Sometimes they are ragged. (Prof. Stephan Borrmann of the Institute of Atmospheric Physics at the Johannes Gutenberg University of Mainz, Germany kindly provided the used cloud pictures.)

Upon microscopic evaluation of CD61 stains, the platelet distribution was interpreted and continuously assigned to the basic meteorological cloud shapes “stratus”, “cirrus”, and “cumulus” (Fig. 2) of the World Meteorological Organization^30^ according to their quantitative occurrence in percentage.

Reading the HE and EvG stains, histopathologists reviewed fibrin nets regarding its quality and assigned percentage values to each of the three features ”fine”, “coarse”, and “dense”. Finally, using the HE stains, neutrophils and red cells were examined assessing their integrity, dividing them up into wholesome or shattered. Continuous percentage scales were used to specify proportions of intact neutrophils and red cells to their total numbers.

According to the pathophysiological hypotheses, we formulated the following THROMBEX classification rule:

The histological structure of an embolus points to an arterioembolic formation process if two of the following three criteria are met:

1. Proportion of dense fibrin ≤ 30%.
2. Platelet distribution meets cumulus criterion ≥ 90%.
3. Proportion of intact neutrophils and red cells ≥ 80%.

Such emboli are defined as “ARTERIO “. Otherwise, histological classification points to a cardioembolic formation process (“CARDIO”).

In terms of an optimization task, the three cut-off values were determined by adjusting the classification rule until the maximum number of the 11 reference thrombi was correctly assigned to their assured pathophysiological formation process.

**Figure 3:**
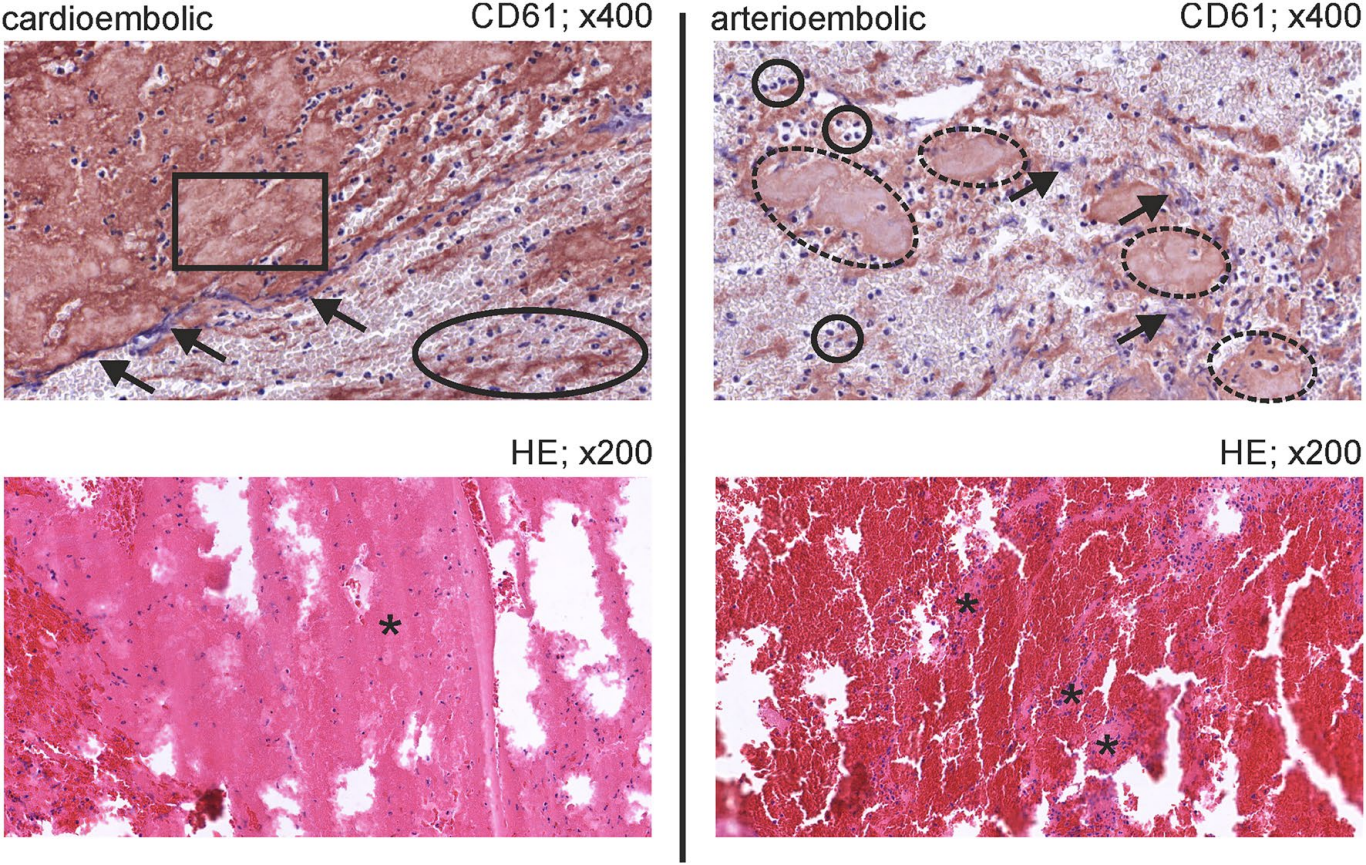
Histological presentation of typical cardioembolic and arterioembolic clots. For the two preparations immunohistochemical staining with CD61 (400-fold original magnification) and hematoxylin–eosin staining (200-fold original magnification) were prepared. The cardioembolic clot (left side) is characterized by a large number of platelets. Their distribution pattern reminds of stratus (rectangle) and cirrus (oval) cloud formations. Many disintegrated neutrophil granulocytes can be seen (arrows). Fibrin net is dense (star). This corresponds to a separation thrombus. In contrast, the arterioembolic clot (right side) is marked by a small amount of platelets arranged like cumulus clouds (dashed ovals). Apart from some disintegrated neutrophils (arrows), many intact neutrophils can be found (circles). Fibrin net is fine to coarse (stars). It is therefore an agglutinative thrombus that presumably arose from the tail part of a mixed-thrombus. Both emboli were extracted from patients with a definite stroke etiology.

Figure 3 shows typical histological specimens of an arterioembolic and a cardioembolic clot that were correctly assigned by the classification rule.

### Calculations and statistics

The proportions of concordant classifications were calculated, and exact 95% confidence intervals (CIs) are provided. Analyses were performed using R version 3.5.0 with the package epitools (version 0.5)^31^.

## Results

Analysis of etiology of the 125 included stroke patients that underwent thrombectomy revealed predominant portions of cardioembolic (n = 55; 44%) and arterioembolic genesis (n = 27; 21.6%) as well as ESUS patients (n = 31; 24.8%). As expected, cases with competing causes (n = 9; 7.2%) or other specific causes (n = 3; 2.4%) were rare.

Only emboli of patients with cardioembolic or arterioembolic strokes were used to validate the classification rule. As expected, the distribution of their baseline characteristics (Table 1) showed that the incidence of cardiovascular risk factors (arterial hypertension, diabetes mellitus, hyperlipoproteinemia, increased body mass index, history of smoking) was rather associated with an arterioembolic genesis of stroke. In contrast, atrial fibrillation was far more frequent in the cardioembolic group (87.3% vs. 3.7%).

Occlusion of the basilar artery occurred almost twice as often in the group of cardioembolic strokes (14.5% vs. 7.4%). No striking difference was found concerning the affected side of the carotid flow area. Severity of stroke symptoms measured by the NIHSS was roughly equal in both groups. Patients with cardioembolic strokes received anticoagulants more often within their premedication. Local application of rt-PA was more frequently used in the course of acute treatment in the cardioembolic group (10.9% vs. 7.4%). No striking differences between the two groups were found regarding systemic thrombolysis or bridging with Abciximab. In the group of cardioembolic strokes, the mean duration between symptom onset and recanalization was shorter (204 vs. 229.5 min), whereas the mean recanalization time was prolonged (52 vs. 48.5 min).

To validate the classification rule agreement between stroke etiology defined by stroke experts and histological evaluation of the extracted emboli performed by two independent histopathologists (rater I and II) was determined. For both raters significant agreement was found (Table 2).

**Table 2:** Fourfold table of the validation data.

Rater I (N = 81)/Rater II (N = 82)	Histological classification	
	CARDIO	ARTERIO
**Stroke etiology**		
Cardioembolic (E1)	39/43	15/12
Arterioembolic (E2)	8/7	19/20
Agreement concerning E1 [%, (95% CI)], Rater I: N = 54; Rater II: N = 55	72,2 (58,4–83,5) / 78,2 (65,0–88,2)	
Agreement concerning E2 [%, (95% CI)], both raters: N = 27	70,4 (49,8–86,3) / 74,1 (53,7–88,9)	
Agreement concerning E1 + E2 [%, (95% CI)], Rater I: N = 81; Rater II: N = 82	71,6 (60,5–81,1) / 76,8 (66,2–85,4)	

Stroke etiology was independently determined by three stroke experts based on complete anamnestic information and diagnostic results concerning cause analysis of stroke. Histological classification was performed by two independent raters that were blinded for the defined etiology of the thrombotic emboli. Values for both raters are indicated before (rater I) and after (rater II) the slash. CI = confidence interval.

Only rater I did not obtain better than chance agreement in the allocation of arterioembolic clots. The histological evaluation of rater II delivered a somewhat higher total agreement (76.8%) than the evaluation of rater I (71.6%). Both raters achieved higher agreement for the classification of cardioembolic clots.

## Discussion

### Determination of stroke etiology and baseline characteristics of the included patients

Three independent stroke experts confirmed in our study the stroke etiology after examining medical history and diagnostic results of all necessary instrumental investigations. Unanimous decision of the three experts was required to specify the underlying etiology of stroke. This etiology was set to be the actual cause of stroke and served as reference to validate the developed classification rule.

The frequencies of the different stroke etiologies obtained (Fig. 1) are quite consistent with results found in the literature^4,32^. Explanations that are more detailed can be found in the discussion section in the Data Supplement.

The baseline characteristics of the included patients (Table 1) reflect some findings concerning already known predictors of different stroke subtypes. As expected, atrial fibrillation and therefore oral anticoagulation is far more frequent in the group of cardioembolic strokes. Atrial fibrillation is the most frequent cause of cardioembolic stroke^33–36^, or is at least a symptom of an endothelial dysfunction that has been recently found to be the main reason for thrombotic emboli^17^. By analogy, there are indications for the already known association between the common cardiovascular risk factors and arteriosclerosis leading to arterioembolic strokes^37–39^. In our patient cohort, basilar artery occlusion was caused more frequently by cardiac embolism. This finding is in line with the results of other work groups^40–42^.

In our study, the mean recanalization time was slightly increased in the cardioembolic group. This is in agreement with a previous study and may suggest a more difficult intervention in cardioembolic stroke^43^. Further considerations are made in the discussion section in the Data Supplement.

Probably, some of the above mentioned baseline characteristics affect the histological composition of the investigated emboli. In particular, medical treatment before symptom onset may play a decisive role.

### Validation of the classification rule

According to the developed classification rule, cardiac emboli comply with separation thrombi with dense fibrin nets, a widespread distribution of platelets, and small proportions of intact neutrophils and red cells. They originate from platelet accumulation caused by an endothelial dysfunction of the heart. These clots are compact, rigid, stable and appear older. Arteriosclerotic emboli arise from agglutinative tail parts of mixed-thrombi or from clotting processes in the slipstream of a stenosis. They are torn away soon after formation by fast streaming blood and flow turbulences. Therefore, they are smooth and loose and their structure is poorly organized. These hypotheses have been developed using pathophysiological considerations and are reflected by the baseline characteristics of our subjects.

The classification rule is successfully validated. Only rater I barely misses significant agreement while assigning arterioembolic clots (Table 2). Therefore, the suggested classification rule is suitable to differentiate between certain cardiac and arteriosclerotic emboli with a prediction accuracy between 70 and 78%.

Both raters identify cardioembolic clots better than arterioembolic clots. This is probably due to the chosen cut-off values because they allocate a larger range for cardioembolic clots. Therefore, classification of cardiac emboli is straightforward.

The accuracy of histological evaluations is naturally limited by pathologist-related, study methodology-related, and specimen-related causes. Therefore, most kappa values concerning reproducibility of histological classifications are in the range of 0.41–0.6 (signifying moderate agreement)^44–50^. For the reliability of WHO and Gleason histologic grading systems in prostatic adenocarcinoma intraobserver reliabilities were 75.0% and 78.1%, respectively. Interobserver reliabilities were 60.4% and 70.8%, respectively^51^. This example shows that even common and useful histological classifications and grading systems are limited concerning their reliability. Considering this, the gained results of our classification rule are valuable keeping in mind that reproducibilities of different studies cannot be compared directly.

### Comparison of the classification rule with results of other working groups

Brinjikji et al. performed a meta-analysis to identify a correlation between clot histology and stroke etiology^10^, identifying studies published between January 2005 and December 2015 that report findings related to histologic and/or imaging characteristics of thrombi in acute ischemic stroke secondary to large vessel occlusion. Finally, nine studies examining the association between the histology and etiology of emboli were selected, including in total 302 stroke patients. The two possible alternatives concerning stroke etiology were cardiac and arteriosclerotic embolism. In five studies, no correlation was found^28,52–55^. Two studies found that cardiac emboli were associated with an increased proportion of red cells and less fibrin^56,57^. In contrast, Niesten et al. found that arteriosclerotic emboli had a significantly higher proportion of red cells combined with cardiac emboli^26^. The research group of Boeckh-Behrens showed that cardiac emboli contained more white blood cells and were characterized by a higher level of formation and organization^43^. Finally, the meta-analysis drew the conclusion that due to insufficient and inconsistent data a correlation cannot be proven. However, it was stressed that most of the underlying histological investigations were done without immunohistochemical stains, therefore lacking important features. It is noteworthy that three^25,26,56^ of four studies using immunohistochemical stains found a correlation between histological features and the etiology of clots.

The classification rule presented fulfills the demand for additional immunohistochemical staining and is in line with the findings of the working groups of Niesten and Boeckh-Behrens^26,43^. Furthermore, it complies with some more recent studies proving that cardiac emboli were significantly correlated with higher proportions of fibrin, platelets, and white blood cells but with a lower proportion of red cells^8,9,16^. Appropriately, two other research groups found a significantly greater red cell proportion in arterioembolic clots than in cardioembolic clots, whereas fibrin proportion was significantly larger in cardioembolic clots that were furthermore characterized by a higher level of organization^7,14^. In a per-pass analysis of embolus composition Duffy et al. found that emboli retrieved in passes 1 and 2 from patients with arterioembolic strokes had a higher red cell composition, whereas the histological characteristics of the overall embolus load removed did not differ significantly among different etiologies^11^. An important limiting factor of their study was that more than one-third of all cases was classified as cryptogenic strokes impeding the correlation between histology of emboli and etiology of strokes.

Contrasting results showing higher red cell proportions^13,15^ and lower fibrin percentages in cardiac emboli^13^ were also obtained. Fitzgerald et al. found significantly higher proportions of platelets for large artery atherosclerosis etiology^12^. Table S1 in the Data Supplement gives an overview of the different studies.

In summary, most of the studies, in particular the both largest studies correspond with our developed classification rule. Further, the majority of studies based on additional immunohistochemical stains leads to concordant results. Most of the studies (4 of 5) contradicting our approach were performed with Asian patients.

All the previous studies are based on group comparison. Our classification rule was developed to enable inferences about stroke causes in individual patients. In particular, this promises huge benefit in strokes that cannot be assigned to a defined cause despite intensive diagnostic workup. Such cases are frequent and bear a high risk of recurrent strokes^57^.

The informative value of all relevant studies in this field is restricted by a single-center design, small numbers of arterioembolic clots, and an inherent selection bias: only those emboli that did not dissolve spontaneously or after rt-PA administration and that can successfully be retrieved via thrombectomy are available for these studies. This impedes the assessment of rt-PA susceptible and thrombectomy-resistant emboli. Furthermore, components of the retrieved embolus might not completely reflect those of the entire embolus. Emboli consist of complex, heterogeneous, and anisotropic materials that undergo a dynamic evolution. In particular medical treatment before symptom onset, the chosen acute therapy, and age of the emboli might alter its histological characteristics. Usually, the number of passes needed to retrieve the occluding embolus is neglected, although the per-pass composition of the clot is likely to change^11^. Finally, it remains uncertain whether the retrieved clot is captured from the exact occlusion site, or from a proximal or distal section.

Discrepancies between different studies may be associated with restrictions in the number of patients, ethnic affiliation of subjects, variations in procedural techniques of endovascular treatment and the lack of standardized methods concerning staining and evaluation of the histological preparations. A consensus statement from experts sought to solidify terminology and establish standardized analysis before conducting a multi-center trial.

### Specific limitations of the present classification rule

In addition to the above-mentioned weaknesses of histological evaluations in general, some specific limitations should be mentioned concerning the present validation process:

The number of reference thrombi is low and finally they are not cerebral emboli. However, their origin is certain making them suitable for the adjustment of the cut-off values. The chosen method to evaluate the embolus includes manual segmentation and personal analysis, which might lead to an observer bias. Although stroke etiology was set unanimously by three independent stroke experts, it is not guaranteed that this cause of stroke is actually true.

Therefore, the present classification rule needs further improvement. In a first step, this could be achieved by more reference thrombi, further adjustment of the cut-off values and a more sophisticated definition of the classification rule.

It should be emphasized that the present study validates the classification rule for patients with a certain cardio- or arterioembolic stroke. Up to now, the accuracy of the present classification rule for embolic strokes of unknown source is not known. Therefore, a further prospective study is planned including patients whose stroke etiology is uncertain at the time of histological evaluation but will be defined later.

## Conclusions

To our knowledge, this is the first attempt to develop a histological classification rule for cerebral emboli based on well-justified pathophysiological considerations and adjustment to reference thrombi before specimens are examined. The present classification rule enables the distinction between cardiac and arteriosclerotic emboli in histological samples of individual stroke patients that underwent thrombectomy by evaluating the fibrin network, distribution of platelets, and the proportion of intact neutrophils and red cells. The classification rule was successfully validated keeping in mind that the accuracy of histological evaluations is naturally restricted. Agreements between histological classification and stroke etiology defined by strokes experts are in the range of 70–78%. Advantages of this histological classification are the absence of harm for the patient and of hindrance for other diagnostic procedures and its easy and cheap implementation.

The present classification rule is supported by most of the relevant results gained by other research groups. Multi-center studies with larger patient numbers should confirm and improve the classification rule. Validation of the present classification rule was successful in cases of ensured cardio- or arterioembolic strokes. To what extent it might indicate the etiology of undetermined embolic strokes remains to be examined in a further prospective study. This is vitally important to improve secondary prevention of patients with embolic strokes of unknown source.

## Supplementary Information

Supplementary Information

## Data Availability

The datasets generated during and/or analysed during the current study are available from the corresponding author on reasonable request.
