# Supplementary material for: First approach to distinguish between cardiac and arteriosclerotic emboli of individual stroke patients applying the histological THROMBEX-classification rule
Source: Sci Rep. 2021 Apr 19;11:8433. doi: 10.1038/s41598-021-87584-2 (PMC8055901; doi:10.1038/s41598-021-87584-2)
Supplement: Supplementary file 1 — Supplementary Information [file 41598_2021_87584_MOESM1_ESM.pdf]

SUPPLEMENTAL MATERIAL

First approach to distinguish between cardiac and arteriosclerotic emboli of individual stroke patients applying the histological THROMBEX-classification rule

Scientific Reports

Florian C. Roessler, MD, PhD[1*]; Nicolas Kalms[1]; Florian Jann[1]; Andrè Kemmling, MD[2]; Julika Ribbat-Idel, MD[3]; Florian Stellmacher, MD[4]; Inke R. König, PhD[5]; Marcus Ohlrich[6]; Georg Royl, MD[7]

[1] Department of Neurology, Justus-Liebig-University Gießen, Klinikstraße 33, 35385 Gießen, Germany.

[2] Department of Neuroradiology, Westpfalz-Klinikum, Hellmut-Hartert-Straße 1, 67655 Kaiserslautern, Germany.

[3] Institute of Pathology, University of Lübeck and University Hospital Schleswig-Holstein, Campus Lübeck, 23538 Lübeck, Germany.

[4] Institute of Pathology, Research Center Borstel - Leibniz Lung Center, 23845 Borstel, Germany.

[5] Institute of Medical Biometry and Statistics, University of Lübeck, Ratzeburger Allee 160 (House 24), 23562 Lübeck, Germany.

[6] Department of Neurology, Sana Kliniken Lübeck GmbH, Kronsforder Allee 71-73, 23560 Lübeck, Germany.

[7] Department of Neurology and Center of brain, behaviour and metabolism, University of Lübeck, Ratzeburger Allee 160, 23538 Lübeck, Germany.

*** Address for correspondence:**

Dr. med. Dr. rer.-medic. Dipl.-Phys. Florian C. Roessler

Klinik und Poliklinik für Neurologie, Universitätsklinikum Standort Gießen, Klinikstraße 33, 35385 Gießen, Germany.

Telephone: 0049-(0)641-985-56814.

E-mail: florian.roessler@neuro.med.uni-giessen.de

Supplemental Discussion

The frequencies of the different stroke etiologies obtained in our study by three independent stroke experts are quite consistent with results found in the literature.

The data indicated by Ntaios et al. enable an accurate comparison [1]: of 2109 patients with complete data and no lacunar stroke, 497 patients (23.6%) met our criteria for an arterioembolic cause of stroke. Furthermore, using our criteria, 869 of their patients (41.2%) would have been assigned to a cardioembolic source of embolism, 275 of their patients (13%) to ESUS, and 102 (4.8%) to other/rare specific causes of stroke. The basic population and workflow of the assignment are not the same in both studies. Nevertheless, the good correlation between the calculated frequencies for cardioembolic and arterioembolic strokes indicate a reasonable determination of stroke etiology by our stroke experts.

In our study, the mean recanalization time was slightly increased in the cardioembolic group. This may suggest a more difficult intervention in cardioembolic stroke.

Sporns et al. found that longer intervention times are associated with a high amount of fibrin [2]. Fibrin-rich clots are firm, tough and sticky, and therefore much less likely to deform challenging endovascular thrombectomy [3-5]. According to our classification rule those clots comply with cardiac emboli.

Furthermore, there are many references indicating that red cell-rich clots that are assigned in our study to an arterioembolic stroke etiology are associated with improved recanalization rates [2-4, 6-11]. This finding coincides with the positive relationship between the hyperdense vessel sign or high clot density on computed tomography, indicative of red cell-rich emboli [8, 10, 12, 13], and shorter procedure times with higher

reperfusion rates [14-17]. Presumably, this might be due to an increased viscosity and elasticity of red cell-rich clots leading to a stronger coherence of thrombus material when subjected to stress by a stent retriever and finally to a better clot retrieval [18-20]. Accordingly, Niesten et al. determined a correlation between the hyperdense artery sign and non-cardioembolic stroke cause [21].

Supplemental Tables

Author, year [reference]	Number of samples	Histological techniques	Result confirms our classification rule
Marder*, 2006 [22]	25 (15 / 4)	HE	0
Liebeskind*, 2011 [12]	50	HE	0
Sato*, 2012 [23]	17 (11 / 6)	HE, PGPIIb/IIIa, glycophorin A and FibII	NO
Niesten*, 2014 [13]	22 (6 / 8)	HE, Mallory´s phosphotungistic acid-hematoxilin, glyophorin A, CD31	YES
Singh*, 2014 [24]	49	HE	0
Kim*, 2015 [25]	37 (22 / 8)	HE, PGPIIIa, CD61	NO
Sallustio*, 2015 [26]	28 (11 / 17)	HE, Mallory´s phosphotungistic acid-hematoxilin	0
Simons*, 2015 [27]	40 (21 / 19)	HE, CD34	0
Ahn, 2016 [28]	36 (22 / 8)	HE, Martius scarlet blue, CD42b	YES
Boeckh-Behrens*, 2016a [6]	34 (16 / 3)	HE, EvG	YES
Boeckh-Behrens, 2016b [7]	136 (67 / 22)	HE	YES
Sporns, 2017 [29]	187 (77 / 35)	HE, EvG, Prussian blue, CD3, CD20, CD68/KiM1P	YES
Berndt, 2018 [30]	32 (19 / 4)	HE	YES
Maekawa, 2018 [10]	43 (30 / 5)	HE	YES
Shin, 2018 [11]	37 (22 / 7)	HE	NO
Duffy, 2019 [31]	60	HE, Martius scarlet blue	YES (concerning passes 1 and 2)
Fitzgerald, 2019 [32]	105	HE, Martius scarlet blue	NO
Gong, 2019 [33]	45 (36 / 9)	HE	NO

Tab. S1 Studies examining the association between histologic composition of emboli and stroke etiology since January 2005 Authors whose studies were considered in the meta-analysis of Brinjikji et al. [8] are marked by a star. Number of samples: The total number of all histologically investigated clots is specified. If known, the numbers of cardioembolic (first number) and arterioembolic clots (second number) are listed in parentheses. Histological techniques (stains): HE = hematoxylin and eosin; PGPIIb/IIIa = platelet glycoprotein IIb/IIIa; glycophorin A = membrane protein expressed on red cells; FibII = antibodies of fibrin; EvG = Elastica-van Gieson; CD3, CD20, CD31, CD34, CD42b, CD61, CD68/KiM1P = different membrane proteins. Results: If results confirm our classification rule, the study is labeled with “YES”, otherwise with “NO”. Studies that did not find any association between histologic clot composition and stroke etiology are labeled with “0”.

Supplemental References

[1] Ntaios, G. et al. Embolic strokes of undetermined source in the Athens stroke registry: a descriptive analysis. *Stroke*. **46**, 176-181 (2015).

[2] Sporns, P.B. et al. Ischemic stroke: histological thrombus composition and pre-interventional CT attenuation are associated with intervention time and rate of secondary embolism. *Cerebrovasc Dis*. **44**, 344-350 (2017).

[3] De Meyer, S.F. et al. Analyses of thrombi in acute ischemic stroke: a consensus statement on current knowledge and future directions. *Int J Stroke*. **12**, 606-614 (2017).

[4] Yuki, I.et al. The impact of thromboemboli histology on the performance of a mechanical thrombectomy device. *AJNR*. **33**, 643-648 (2012).

[5] Gunning, G.M., McArdle, K., Mirza, M., Duffy, S., Gilvarry, M., Brouwer, P.A. Clot friction variation with fibrin content; implications for resistance to thrombectomy. *J Neurointerv Surg*. **10**, 34-38 (2018).

[6] Boeckh-Behrens, T. et al. The impact of histological clot composition in embolic stroke. *Clin Neuroradiol.* **26**, 189-197 (2016).

[7] Boeckh-Behrens, T. et al. Thrombus histology suggests cardioembolic cause in cryptogenic stroke. *Stroke*. **47**, 1864-1871 (2016).

[8] Brinjikji, W. et al. Correlation of imaging and histopathology of thrombi in acute ischemic stroke with etiology and outcome: a systematic review. *J Neurointerv Surg*. **9**, 529-534 (2017).

[9] Hashimoto, T. et al. Histopathologic analysis of retrieved thrombi associated with successful reperfusion after acute stroke thrombectomy. *Stroke*. **47**, 3035-3037 (2016).

[10] Maekawa, K. et al. Erythrocyte-rich thrombus is associated with reduced number of maneuvers and procedure time in patients with acute ischemic stroke undergoing mechanical thrombectomy. *Cerebrovasc Dis Extra*. **8**, 39-49 (2018).

[11] Shin, J.W., Jeong, H.S., Kwon, H.J., Song, K.S., Kim, J. High red blood cell composition in clots is associated with successful recanalization during intra-arterial thrombectomy. *PLoS One*. doi:10.1371/journal.pone.0197492 (2018).

[12] Liebeskind, D.S. et al. CT and MRI early vessel signs reflect clot composition in acute stroke. *Stroke*. **42**, 1237-1243 (2011).

[13] Niesten, J.M. et al. Histopathologic composition of cerebral thrombi of acute stroke patients is correlated with stroke subtype and thrombus attenuation. *PLoS One*. 9:e88882 (2014).

[14] Brekenfeld, C., Gralla, J., Zubler, C., Schroth, G. Mechanical thrombectomy for acute ischemic stroke. *Rofo*. **184**, 503-512 (2012).

[15] Froehler, M.T. et al. The hyperdense vessel sign on CT predicts successful recanalization with the Merci device in acute ischemic stroke. *J Neurointerv Surg*. **5**, 289-293 (2013).

[16] Moftakhar, P. et al. Density of thrombus on admission CT predicts revascularization efficacy in large vessel occlusion acute ischemic stroke. *Stroke*. **44**, 243-245 (2013).

[17] Mokin, M. et al. Thrombus density predicts successful recanalization with Solitaire stent retriever thrombectomy in acute ischemic stroke. *J Neurointerv Surg*. **7**, 104-107 (2015).

[18] Gersh, K.C., Nagaswami, C., Weisel, J.W. Fibrin network structure and clot mechanical properties are altered by incorporation of erythrocytes. *Thromb Haemost*. **102**, 1169-1175 (2009).

[19] Yoo, A.J., Andersson, T. Thrombectomy in acute ischemic stroke: challenges to procedural success. *J Stroke*. **19**, 121-130 (2017).

[20] Wohner, N. Role of cellular elements in thrombus formation and dissolution. *Cardiovasc Hematol Agents Med Chem*. **6**, 224-228 (2008).

[21] Niesten, J.M. et al. DUtch acute Stroke Trial (DUST). Relationship between thrombus attenuation and different stroke subtypes. *Neuroradiology*. **55**, 1071-1079 (2013).

[22] Marder, V.J. et al. Analysis of thrombi retrieved from cerebral arteries of patients with acute ischemic stroke. *Stroke*. **37**, 2086-2093 (2006).

[23] Sato, Y. et al. Thrombus components in cardioembolic and atherothrombotic strokes. *Thrombosis Research*. **130**, 278-280 (2012).

[24] Singh, P., Doostkam, S., Reinhard, M. Cerebrovascular occlusive disease histopathological analysis of thrombi retrieved by endovascular mechanical extraction during acute ischemic stroke. *Int J Stroke*. **9**, 143 (2014).

[25] Kim, S.K., Yoon, W., Kim, T.S., Kim, H.S., Heo, T.W., Park, M.S. Histologic analysis of retrieved clots in acute ischemic stroke: correlation with stroke etiology and gradient-echo MRI. *AJNR*. **36**, 1756-1762 (2015).

[26] Sallustio, F. et al. Histological features of intracranial thrombo-emboli predict response to endovascular therapy for acute ischemic stroke. *J Neurological Disorders & Stroke*. **3**, 1105 (2015).

[27] Simons, N., Mitchell, P., Dowling, R., Gonzales, M., Yan, B. Thrombus composition in acute ischemic stroke: a histopathological study of thrombus extracted by endovascular retrieval. *J Neuroradiol*. **42**, 86-92 (2015).

[28] Ahn, S.H. et al. Histologic features of acute thrombi retrieved from stroke patients during mechanical reperfusion therapy. *Int J Stroke*. **11**, 1036-1044 (2016).

[29] Sporns, P.B. et al. Ischemic stroke: what does the histological composition tell us about the origin of the thrombus? *Stroke*. **48**, 2206-2210 (2017).

[30] Berndt, M. et al. Thrombus permeability in admission computed tomographic imaging indicates stroke pathogenesis based on thrombus histology. *Stroke*. **49**, 2674-2682 (2018).

[31] Duffy, S. et al. Per-pass analysis of thrombus composition in patients with acute ischemic stroke undergoing mechanical thrombectomy. *Stroke*. **50**, 1156-1163 (2019).

[32] Fitzgerald, S. et al. Platelet-rich emboli in cerebral large vessel occlusion are associated with a large artery atherosclerosis source. *Stroke*. **50**, 1907-1910 (2019).

[33] Gong, L. et al. Bridging therapy versus direct mechanical thrombectomy in patients with acute ischemic stroke due to middle cerebral artery occlusion: a clinical-histological analysis of retrieved thrombi. *Cell Transplant*. doi:10.1177/0963689718823206 (2019).
